# Association of lung recruitment and change in recruitment-to-inflation ratio from supine to prone position in acute respiratory distress syndrome

**DOI:** 10.1186/s13054-023-04428-3

**Published:** 2023-04-13

**Authors:** Lorenzo Del Sorbo, Manuel Tisminetzky, Lu Chen, Laurent Brochard, Daniel Arellano, Roberto Brito, Juan C. Diaz, Rodrigo Cornejo

**Affiliations:** 1 Interdepartmental Division of Critical Care Medicine, University of Toronto, Toronto, Canada; 2 Department of Medicine, Division of Respirology, University Health Network/Sinai Health System, University of Toronto, Toronto, Canada; 3 Keenan Research Centre and Li Ka Shing Institute, Department of Critical Care, St. Michael’s Hospital, Unity Health Toronto, Toronto, Canada; 4 Unidad de Pacientes Criticos, Departamento de Medicina, Hospital Clinico Universidad de Chile, Dr. Carlos Lorca Tobar 999, Independencia, Santiago Chile; 5 Departamento de Radiología, Hospital Clínico Universidad de Chile, Santiago, Chile; 6 Toronto General Hospital, 585 University Avenue, MaRS Centre 9-9021, Toronto, ON M5G 2N2 Canada

**Keywords:** Acute respiratory distress syndrome, Prone positioning, Recruitment-to-inflation ratio, Lung recruitment

## Abstract

Prone positioning is an evidence-based treatment for patients with moderate-to-severe acute respiratory distress syndrome. Lung recruitment has been proposed as one of the mechanisms by which prone positioning reduces mortality in this group of patients. Recruitment-to-inflation ratio (R/I) is a method to measure potential for lung recruitment induced by a change in positive end-expiratory pressure (PEEP) on the ventilator. The association between R/I and potential for lung recruitment in supine and prone position has not been studied with computed tomography (CT) scan imaging. In this secondary analysis, we sought to investigate the correlation between R/I measured in supine and prone position with CT and the potential for lung recruitment as measured by CT scan. Among 23 patients, the median R/I did not significantly change from supine (1.9 IQR 1.6–2.6) to prone position (1.7 IQR 1.3–2.8) (paired *t* test *p* = 0.051) but the individual changes correlated with the different response to PEEP. In supine and in prone position, R/I significantly correlated with the proportion of lung tissue recruitment induced by the change of PEEP. Lung tissue recruitment induced by a change of PEEP from 5 to 15 cmH_2_O was 16% (IQR 11–24%) in supine and 14.3% (IQR 8.4–22.6%) in prone position, as measured by CT scan analysis (paired *t* test *p* = 0.56). In this analysis, PEEP-induced recruitability as measured by R/I correlated with PEEP-induced lung recruitment as measured by CT scan, and could help to readjust PEEP in prone position.

## Introduction

Prone positioning is an established treatment for patients with moderate-to-severe acute respiratory distress syndrome (ARDS) resulting in a significant survival benefit [1, 2]. Several studies suggest that this benefit derives from reduction of ventilator-induced lung injury (VILI), rather than improvement in gas exchange [3]. Lung recruitment has been identified as one of the mechanisms by which prone positioning reduces VILI [4].

Recently, Chen et al. described the recruitment-to-inflation ratio (R/I), a method to measure potential for lung recruitment induced by a change in positive end expiratory pressure (PEEP) [5]. Interestingly, Cour et al. showed that the change of R/I from supine to prone position in patients with COVID-19 related ARDS significantly correlated to changes of the compliance of the respiratory system. In high recruiters (R/I above median), the improvement of compliance, oxygenation and ventilatory ratio in prone position compared to the supine baseline measurement was associated with a reduction of R/I, suggesting less recruitability in prone position due to true lung recruitment. In low recruiters (R/I below median), only oxygenation improved in prone compared to the supine position, without significant changes in R/I [6]. Nonetheless, the association between R/I and potential for lung recruitment in supine and prone position has never been explored with computed tomography (CT) scan imaging.

Cornejo et al. studied in 24 patients with ARDS the potential for lung recruitment in supine and prone position by analysis of CT scan imaging performed at PEEP of 5, 15 and 45 cmH_2_O. CT scans were performed within 30 min of position change [4]. The results demonstrated that prone position significantly increases lung recruitment and reduces high PEEP-induced alveolar hyperinflation. In this study, the measurements of the respiratory system compliance and the CT scan-based lung volume at PEEP of 5 and 15 cmH_2_O allows to retrospectively calculate a CT scan-based R/I. This is calculated using the original R/I equation and replacing the bedside measurement of the difference of end expiratory lung volume between PEEP-high and PEEP-low with the difference of gas volumes measured by CT scan between PEEP of 15 and 5 cmH_2_O, according to the following equation: [5, 7]

$$\begin{aligned}&{\text{Gas volume}}\;\left( {{\text{ml}}} \right) \\ &\quad = {\text{CT number}}\;\left( {\text{Hounsfield Units}} \right)/ - 1000\\ &\quad \times {\text{total volume}}.\end{aligned}$$

- R/I = *C*_rec_/*C*_rs_ at PEEP_5cmH2O_
- *C*_rec_ = DeltaV_rec_/(PEEP_high_ − PEEP_low_)
- DeltaV_rec_ = Delta end expiratory lung volume between PEEP_high_ and PEEP_low_ − Predicted Delta end expiratory lung volume
- Delta end expiratory lung volume between PEEP_high_ and PEEP_low_ = CT gas volume PEEP_15cmH2O_ − CT gas volumePEEP_5cmH2O_
- Predicted Delta end expiratory lung volume = *C*_rs_ at PEEP_5cmH2O_ × (PEEP_15cmH2O_ − PEEP_5cmH2O_) = *C*_rs_ at PEEP_5cmH2O_ × 10 cmH_2_O
- PEEP_high_ − PEEP_low_ = PEEP_15cmH2O_ − PEEP_5cmH2O_ = 10 cmH_2_O
- *C*_rs_ at PEEP_5cmH2O_ = compliance of the respiratory system at PEEP of 5 cmH_2_O

Therefore, we performed a secondary analysis of the data from the study by Cornejo et al. to investigate whether R/I measured in in supine and prone position correlates with potential for lung recruitment, as measured by CT scan. [4]

## Methods

We calculated the CT scan-based R/I for each patient of the study in supine and prone position as described above. The distribution of the variables of interest is expressed as median and interquartile range (IQR).

To calculate PEEP-induced lung recruitment by CT scan we used the method described by Chiumello et al. measuring the proportion of total lung weight of noninflated (NAT) and poorly inflated (PAT) tissue recruited when changing PEEP from 5 to 15 cmH_2_O, according to the following equation: [7]

$$\begin{aligned}&{\text{lung tissue recruitment}} = 100 \\ &\quad \times \left\{ [({\text{NAT}} + {\text{PAT}})\;{\text{weight}}/{\text{total lung weight}}]_{{{\text{PEEP5}}}} \right.\\ &\quad \left.- [({\text{NAT}} + {\text{PAT}})\;{\text{weight}}/{\text{total lung weight}}]_{{{\text{PEEP15}}}} \right\}. \end{aligned}$$

To evaluate the relationship between R/I and lung recruitment as measured by CT scan we used Rho Spearman correlation. *R*^2^ is shown as a measure of model fit.

## Results

Twenty-three out of twenty-four subjects in the original study were included in this reanalysis. One subject was excluded as a CT scan was missing and precluded our reanalysis.

**Fig. 1:**
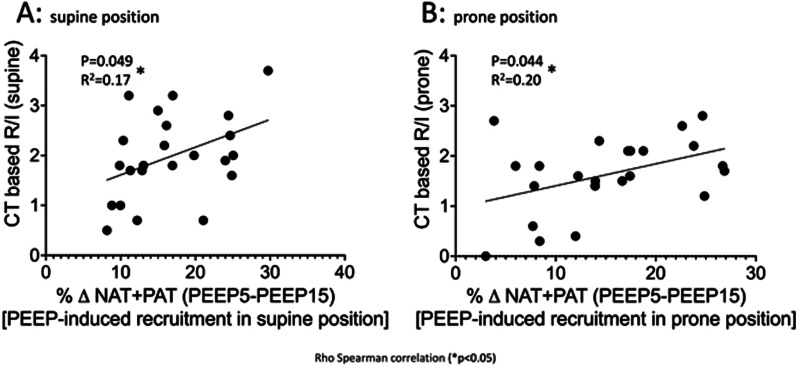
Association between R/I and lung recruitment as measured by CT scan analysis, in supine (**A**) and prone (**B**) position. NAT = Non aerated tissue; PAT = poorly aerated tissue; PEEP = positive end-expiratory pressure

The median R/I did not significantly change from supine (1.9 IQR 1.6–2.6) to prone position (1.7 IQR 1.3–2.8) (paired *t* test *p* = 0.051). The proportion of lung tissue recruitment induced by the change of PEEP from 5 to 15 cmH_2_O was 16% (IQR 11–24%) in supine and 14.3% (IQR 8.4–22.6%) in prone position, as measured by CT scan analysis (paired *t* test *p* = 0.56). In supine (Fig. 1A) and in prone (Fig. 1B) position, the R/I significantly correlated with the proportion of lung tissue recruitment induced by the change of PEEP.

**Fig. 2:**
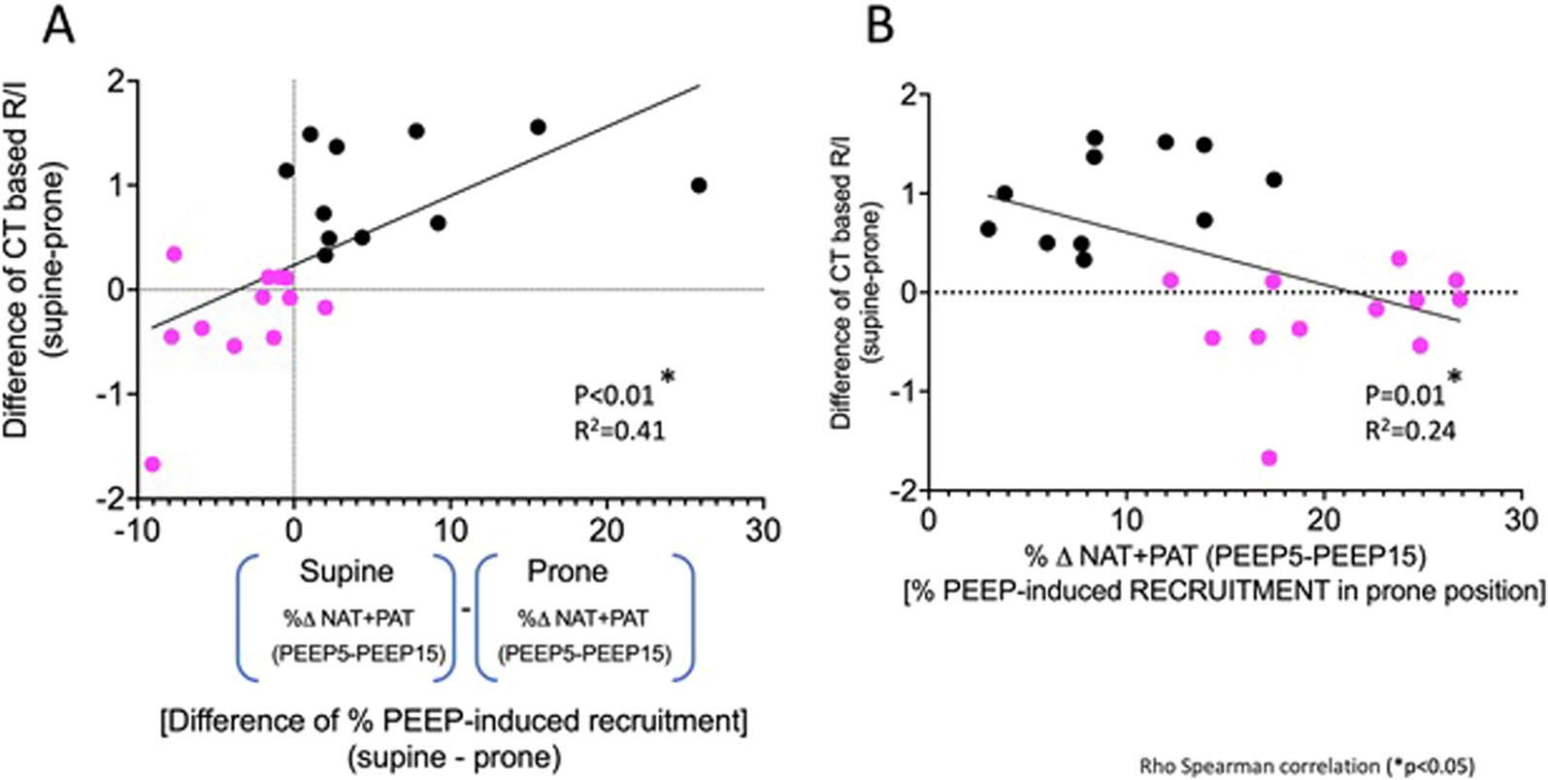
**A** Association between ΔR/I (difference of R/I_supine_ − R/I_prone_) and the difference of the percentage of PEEP-induced recruitment between supine and prone position, as measured by CT scan. Patients were mainly distributed in two quadrants. In the left lower quadrant, pink dots represent the group of patients in which recruitability is higher in prone compared to supine position (negative ΔR/I). In these patients, PEEP-induced recruitment, as measured by CT scan, was also higher in prone than supine position. In the right upper quadrant, black dots represent the group of patients with a positive ΔR/I, indicating that PEEP-induced recruitability is lower in prone compared to supine position. **B**: Association between ΔR/I and PEEP-induced recruitment in prone position. The group of patients color-coded in panel A show in panel **B** lower recruitment induced by PEEP in prone position, suggesting that lung recruitment has already occurred due to the turn in prone position

We used the ΔR/I (R/I_supine_ − R/I_prone_) to express the individual change in recruitability between prone and supine position. This value significantly correlated with the difference in PEEP-induced lung recruitment in prone compared to supine position, as measured by CT scan (supine_PEEP-induced lung tissue recruitment%_ − prone_PEEP-induced lung tissue recruitment%_) (Fig. 2A). When these two values were plotted, patients were mainly distributed in two quadrants. In the left lower quadrant, patients (pink dots) have a negative ΔR/I, indicating that recruitability is higher in prone compared to supine position. In these patients, PEEP-induced recruitment, as measured by CT scan, was also higher in prone than supine position. In the right upper quadrant, patients (black dots) have a positive ΔR/I, indicating that PEEP-induced recruitability is lower in prone compared to supine position. In these patients, PEEP-induced recruitment, as measured by CT scan, was also higher in supine than prone position, suggesting that the reduced recruitability in prone position is the result of a true lung recruitment. Indeed, the ΔR/I inversely correlated with the portion of tissue lung recruited from PEEP 5 to 15 cmH_2_O in prone position (Fig. 2B); therefore, the higher the ΔR/I, the lower is the recruitment induced by PEEP in prone position.

## Discussion

Our study demonstrated that PEEP-induced recruitability as measured by R/I significantly correlates with PEEP-induced lung recruitment as measured by CT scan, in both prone and supine position. The correlation is stronger in prone position compared to supine position, which could potentially be explained by the lower pleural pressure gradient in prone position, resulting in more homogeneous distribution of aeration, and by undetected higher airway opening pressure in supine position, which results in underestimation of the respiratory system compliance. Our results show that the calculation of the ΔR/I between supine and prone position may enable the identification of patients with different recruitment response to PEEP when in prone position. Patients with negative ΔR/I have higher PEEP-induced recruitment while in prone position and hence may benefit from a high PEEP strategy. Conversely, patients with positive ΔR/I, in whom lung recruitment has already occurred due to the turn in prone position, may require low levels of PEEP.

Our study has important limitations. First, it is a secondary analysis of a study with a small sample size, and we could only reanalyze twenty-three out of twenty-four subjects on the original study. Second, patients were recruited only in one centre. Third, for the calculation of R/I lung volumes was not measured from the ventilator at the bedside but calculated from analysis of CT scan imaging in static conditions, which may overestimate gas volumes [8].

In conclusion, measurement of R/I in supine and prone position correlates with CT scan-assessed lung recruitability and may be instrumental in investigating the heterogeneity of treatment effect across individuals receiving prone position.

## Data Availability

The datasets and materials used and/or analyzed during the current study are available from the corresponding author on reasonable request.

## Abbreviations

VILI: Ventilator-induced lung injury
ARDS: Acute respiratory distress syndrome
R/I: Recruitment-to-inflation ratio
PEEP: Positive end-expiratory pressure
CT: Computed tomography
